# Stem Cell Therapy in Limb Ischemia: State-of-Art, Perspective, and Possible Impacts of Endometrial-Derived Stem Cells

**DOI:** 10.3389/fcell.2022.834754

**Published:** 2022-05-23

**Authors:** Saeed Khodayari, Hamid Khodayari, Somayeh Ebrahimi-Barough, Mehdi Khanmohammadi, Md Shahidul Islam, Miko Vesovic, Arash Goodarzi, Habibollah Mahmoodzadeh, Karim Nayernia, Nasser Aghdami, Jafar Ai

**Affiliations:** ^1^ Tissue Engineering and Applied Cell Sciences, School of Advanced Technologies in Medicine, Tehran University of Medical Science, Tehran, Iran; ^2^ Breast Disease Research Center, Tehran University of Medical Sciences, Tehran, Iran; ^3^ International Center for Personalized Medicine (P7MEDICINE), Düsseldorf, Germany; ^4^ Department of Regenerative Medicine, Cell Science Research Center, Royan Institute for Stem Cell Biology and Technology, ACECR, Tehran, Iran; ^5^ Skull Base Research Center, The Five Senses Institute, School of Medicine, Iran University of Medical Sciences, Tehran, Iran; ^6^ Department of Mathematics, Statistics, and Computer Science, University of Illinois at Chicago, Chicago, IL, United States; ^7^ Department of Infectious Diseases and Tropical Medicines, Tehran University of Medical Sciences, Tehran, Iran

**Keywords:** stem cell therapy, limb ischemia, state-of-art, perspective, molecular mechanism, endometrial-derived stem cells, regeneration

## Abstract

As an evidence-based performance, the rising incidence of various ischemic disorders has been observed across many nations. As a result, there is a growing need for the development of more effective regenerative approaches that could serve as main therapeutic strategies for the treatment of these diseases. From a cellular perspective, promoted complex inflammatory mechanisms, after inhibition of organ blood flow, can lead to cell death in all tissue types. In this case, using the stem cell technology provides a safe and regenerative approach for ischemic tissue revascularization and functional cell formation. Limb ischemia (LI) is one of the most frequent ischemic disease types and has been shown to have a promising regenerative response through stem cell therapy based on several clinical trials. Bone marrow-derived mononuclear cells (BM-MNCs), peripheral blood CD34-positive mononuclear cells (CD34^+^ PB-MNCs), mesenchymal stem cells (MSCs), and endothelial stem/progenitor cells (ESPCs) are the main, well-examined stem cell types in these studies. Additionally, our investigations reveal that endometrial tissue can be considered a suitable candidate for isolating new safe, effective, and feasible multipotent stem cells for limb regeneration. In addition to other teams’ results, our in-depth studies on endometrial-derived stem cells (EnSCs) have shown that these cells have translational potential for limb ischemia treatment. The EnSCs are able to generate diverse types of cells which are essential for limb reconstruction, including endothelial cells, smooth muscle cells, muscle cells, and even peripheral nervous system populations. Hence, the main object of this review is to present stem cell technology and evaluate its method of regeneration in ischemic limb tissue.

## Introduction

Peripheral arterial disease (PAD) results in a wide range of organ dysfunction and organ failure in humans. It has been reported that over 200 million PAD cases had been diagnosed just in 2017 globally (Fowkes et al., 2017). Meanwhile, limb ischemia (LI) in particular leads to a large number of these types of disorders (Torbjörnsson et al., 2021). Among these, acute limb ischemia (ALI) and critical limb ischemia (CLI) are considered the most common diagnostic cases of LI, which occur when there is a sudden lack of circulation to limbs due to occlusion of a peripheral artery or bypass graft (Shoji et al., 2020; Torbjörnsson et al., 2021). Through limb ischemia, peripheral arterial disorders, atherosclerotic peripheral vascular disease, and embolic occlusion can directly arise by reducing the blood perfusion into the limb tissues (Narula et al., 2018). Pain in the ischemic regions, thickening of the toenails, skin infections, and limb ulcers are considered the main symptoms of LI (Lambert and Belch, 2013). Interventions such as vascular and endovascular surgery are commonly used as standard approaches to help regulate and promote circulation to ischemic limbs (Almasri et al., 2019). Despite prescribed treatments, a significant number of patients with LIs have to undergo a major lower limb amputation (Fowkes et al., 2017); hence, the development of a safe, minimally invasive, and effective strategy for regenerating degenerated tissues is regarded as the primary strategy for the treatment of the LI.

Nowadays, the undeniable advantage of using stem cell technology is widely acknowledged as a reliable, safe, and effective method for generating mature cells (Khodayari et al., 2021a; Khodayari et al., 2021b) and regenerating injured tissues and organs (Emadedin et al., 2012; Rigato et al., 2017; Khodayari et al., 2019; Hashemian et al., 2021). From a developmental and physiological view throughout the animal’s lifetime, stem cells play a vital role in the hemostasis, healing, and regeneration of the organs (Ghazizadeh et al., 2018; Khodayari et al., 2019; Beeken et al., 2021). Based on diverse experimental and clinical studies, stem cell-based therapies have been deemed suitable for managing the damaged limb tissues and improving the LI’s symptoms (Zafarghandi et al., 2010; Molavi et al., 2016; Gao et al., 2019).

So far, various types of multipotent stem/progenitor cells have been successfully tested as potential candidates for limb regeneration. The therapeutic potential of BM-MNCs, MSCs, CD34^+^ MNCs, ESPCs, EnSCs, and neural stem cells (NSCs) has been tested on LI patients with different criteria (Supplementary Table S1). Our clinical trials have shown promising results in terms of safety, long-term advantages, and feasibility of the BM-MNC and MSC therapies for limb tissue regeneration/reconstruction targets (Zafarghandi et al., 2010; Emadedin et al., 2012; Molavi et al., 2016). Promoting neovascularization, myogenesis, and neurogenesis, as well as secretion of different paracrine/autocrine factors into the injury microenvironment (Table 1), would be the main therapeutic benefit for the damaged limbs resulting from these transplanted stem cells. In this case, inducing therapeutic angiogenesis into the ischemic regions is the chief regenerative mechanism that is promoted by the stem cells (Johnson et al., 2019). This therapeutic response is triggered through the stem cell differentiation to endothelial cells and secreting various types of pro-angiogenic factors into the injured tissue microenvironment (Khodayari et al., 2019). Based on experimental observations, the MSCs, EPCs, and EnSCs are the cells that show both the responses after transplantation (Shamosi et al., 2015; Bouland et al., 2021), while the MNCs have no significant differentiation potential, and it seems their efficacy is induced *via* regulating the ischemic tissue environment (Li et al., 2006). These facts raise the possibility that releasing pro-angiogenic factors can be the main factor for inducing therapeutic angiogenesis into the injured limb.

**TABLE 1:** Characteristics, biological activity, and main clinical applications of the approved stem/progenitor cell types for treatment of limb ischemia.

Stem cell type	Developmental origin	Sources	Phenotype	Regeneration potential/generated linages	Paracrine/autocrine factors	Other clinical applications	References
BM-MNCs	Non-hematopoietic origins such as ectoderm, mesoderm, and endoderm	Bone marrow	CD34^+^, PROM1 (CD133)^+^, KIT (C-Kit)^+^, CD14^−^, and CD45^−^	Myogenesis, angiogenesis, osteogenesis, as well as hepatogenesis, and neural lineage differentiation	Cytokines and immune suppressors: NOS, IL-8, IL-10, and TGF-β	Cardiovascular disorders	Heldman et al. (2014)
					Growth factors: EGF, PDGF, VEGF, and SDF-1		
					Chemokine/surface markers: CXCL8, CXCL5, CXCL1, and CCL5		
						Diabetes and its related complications	(Dubsky et al., 2013; Wu et al., 2014)
						Brain diseases and spinal cord injuries	(Sarasúa et al., 2011; Moniche et al., 2015; Liu et al., 2017)
						Bone fracture and disease	Seebach et al. (2016)
						Skin regeneration	Zhou et al. (2016)
						Liver disease	Mohamadnejad et al. (2016)
MSCs	Somatic lateral plate mesoderm	Bone marrow, peripheral blood, umbilical cord blood/tissue, as well as adipose, muscle, skin, and cardiac tissues	CD73^+^, CD90^+^, 105^+^, CD34^−^, CD45^−^, D11b^−^, CD14^−^, CD19^−^, CD79a^−^, and HLA-DR^-^	Myogenesis, angiogenesis, osteogenesis, ligament and tendogenesis as well as hepatogenesis, dipogenesis, and neural lineage differentiation	Cytokines/immune suppressors: NO, IL-10, TGF-β, and CCL-2	Cardiovascular disorders	Kim et al. (2018)
					Growth factors: EGF, PDGF, VEGF, BDNF, IGF-1 and SDF-1		
					Chemokines/surface markers: galectin, ICAM-1, and VCAM-1		
						Diabetes	Bhansali et al. (2017)
						Brain and spinal cord injuries	(Xiao et al., 2018; Levy et al., 2019)
						Multiple system atrophy	Singer et al. (2019)
						Pulmonary and respiratory diseases	Wilson et al. (2015)
						Hepatic disorders	Shi et al. (2012)
						Bone fracture and disease	Emadedin et al. (2018)
						Kidney disease	(Makhlough et al., 2017; Makhlough et al., 2018)
						Autoimmune disease	Liang et al. (2018)
						Skin regeneration	Kim et al. (2020)
PB-CD34^+^ MNCs	Non-hematopoietic origins such as ectoderm, mesoderm, and endoderm	Peripheral blood, umbilical cord blood, and bone marrow	CD34^+^, CD14^−^, and CD45^−^	Myogenesis, angiogenesis, osteogenesis, as well as hepatogenesis, and neural lineage differentiation	Cytokines and immune suppressors: SGPC, PGPC, NOS, IL-8, IL-10, and TGF-β	Cardiovascular disorders	Tendera et al. (2009)
					Growth factors: EGF, PDGF, VEGF, and SDF-1, and BDNF		
					Chemokine/surface markers: CXCL8, CXCL5, CXCL1, and CCL5		
						Hepatic disorders	Park et al. (2013)
						Hematopoietic recovery flowing chemotherapy	Cancelas et al. (1998)
ESPCs/NMPB-ACPs	Endothelial and hematopoietic cell lineages	Peripheral blood, umbilical cord blood/tissue, hemogenic endothelium, as well as adipose, muscle, skin, and cardiac tissues	CD34^+^, CD133^+^, ANGPT2+, CD144+, VEGFR^+^, GATA2^+^, PDGFB^+^, CD31^+^, CD14^−^, and KDR^-^	Neovascularizationa and re-endothelialization	Cytokines and immune suppressors: IL-10, eNOS, and TGF-β	Cardiovascular disorders	(Lee et al., 2015; Zhu et al., 2016)
					Growth factors: VEGF-A, VEGF-B, SDF-1, and IGF-1	Osteonecrosis	Daltro et al. (2015)
					Chemokine and surface markers: CXCR-4 and VCAM-1	Diabetes and its related complications	Tanaka et al. (2014)
CTX/NSCs	Ectoderm	Brain and spinal cord SVZ, oncogene immortalized stem cells, neurospheres, and embryonic stem cell (ES)-derived neural cells	CD184^+^, CD24^+^, nestin^+^, FGF-R^+^, GFAP^+^, SOX1/2^+^, FOXO-3^+^, TLX^+^, CD271^-^, and CD44^−^	Neural lineage differentiation	Growth factors: EGF, b-FGF, IGF-1, VEGF, GDNF, NGF, and BDNF	Brain and spinal cord injuries	(Kalladka et al., 2016; Curtis et al., 2018)
						Brain cancer treatment	Portnow et al. (2017)
						Peripheral arterial disease	
EnSCs/ERCs	Mesoderm	Endometrium	CD146^+^, PDGF-Rβ^+^, CD29^+^, CD44^+^, CD73^+^, CD90^+^, CD105^+^, SSEA-1^-^, CD34^−^, CD31^−^, and CD45^−^	Myogenesis, angiogenesis, osteogenesis, ligament and tendogenesis as well as hepatogenesis, adipogenesis, and neural lineage differentiation	Cytokines/immune suppressors: NO, IL-10, TGF-β, and CCL-2	Cardiovascular disorders	Bockeria et al. (2013)
					Growth factors: EGF, PDGF, VEGF, BDNF, IGF-1, and SDF-1	Peripheral vascular diseases	Zhong et al. (2009)
					Chemokines/surface markers: galectin, ICAM-1, and VCAM-1		

The EnSCs’ biology, differentiation, and regeneration potential have been thoroughly studied during our investigative process. In this regard, we could track the neurogenic, angiogenic, and myogenic potential of the mouse and human EnSCs in both *in vitro* and *in vivo* conditions (Ai and Ebrahimi, 2010; Mobarakeh et al., 2012; Tabatabaei et al., 2013; Khademi et al., 2014; Tehrani et al., 2014; Shamosi et al., 2017). In addition to the basic and clinical observations we have summarized in Table 1, Supplementary Table S1, our studies could reliably conclude that the EnSCs may be a novel and useful source for regenerating various types of ischemic disorders, along with other stem/progenitor cells (Ai and Ebrahimi, 2010).

Although different clinical examinations report the benefits of cell therapy on the ischemic organs, there are multiple challenges and barriers to achieving satisfactory regeneration in practice. In 2019, Khodayari et al. (2019) hypothesized that the creation of a stressful inflammatory microenvironment following the acute phase of myocardial infarction can be a potential inherent factor in blocking the outcome of cell therapy in this ischemic region. We believe creating this type of natural restriction would be a destructive factor for stem cell therapy goals, not only for infarcted myocardium but also for all other types of ischemic disorders. It seems that 1) developing modern technologies to reduce the cost of manufacturing stem cells at the highest level, 2) creating next-generation stem cells compatible with the ischemic microenvironment, and 3) presenting new available stem cell sources with greater regenerative potential will be the adequate targets to achieve more efficient tissue regeneration in practice. The scope of this review article is to first describe the molecular mechanisms of pathogenesis of limb ischemia. After summarizing our clinical experiences and the other latest clinical trials on LI stem cell therapy, we will present our group’s obtained evidence that could illustrate the EnSCs’ therapeutic impacts on limb regeneration. Finally, we will outline the limitations and perspectives of the cell therapy approach for the regeneration of limb ischemia.

## Limb Ischemia Pathogenesis: Pathways and Mechanisms

Based on recent studies, peripheral arterial disorder is the main factor in the progression of hind limb ischemia (HLI). Chronic and acute arterial occlusions are caused primarily by occlusive arterial diseases and could block the blood perfusion into the limbs, as well as related tissues and organs (Heo et al., 2017). During the ischemia pathogenesis, decreasing the hypoxic cell’s metabolism, increasing the level of intracellular free radicals, and forming a stressful inflammatory network in the ischemic tissue microenvironment can negatively disturb the cellular population’s viability and function (Khodayari et al., 2019).

**FIGURE 1:**
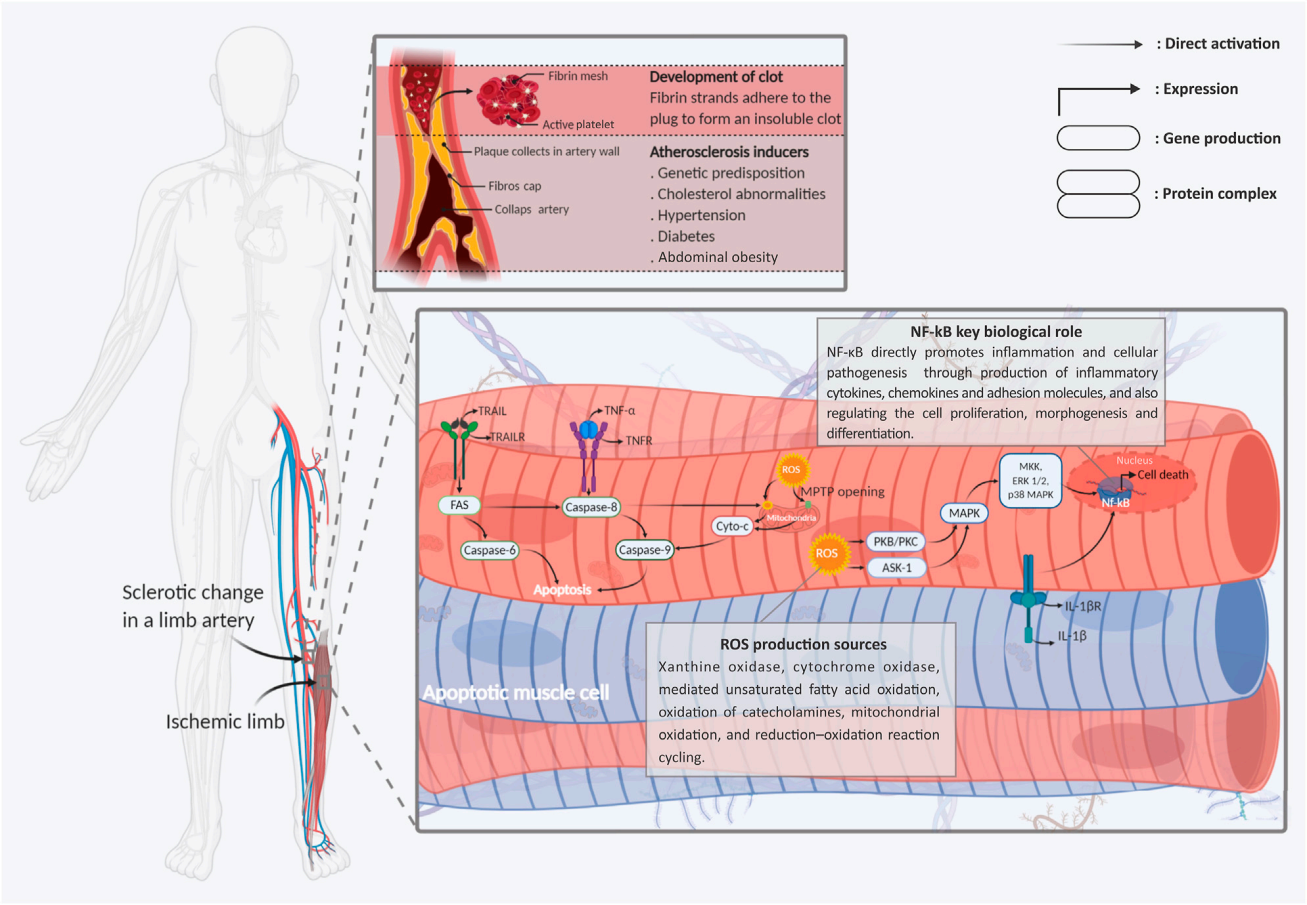
Schematic representation of the main molecular mechanisms involved in limb ischemic pathogenesis. Two types of apoptotic skeletal muscle cells with fragmented nuclei under hypoxic stress and skeletal muscle cells are shown. Following ischemia, endogenic ROS production is induced early after injuries. Cytoplasmic ROS *via* chains of mPTPs opening of the mitochondrial membrane, releasing of the cyto c into the cells’ cytoplasm, and activation and intra-nucleus accumulation of Nf-kB through MAPK signaling stimulation, leads to the muscle cells’ apoptosis and necrosis. In addition to, stimulation of the TNF-R1/2, IL-1R, and TRAIL-R, as the main death ligands, by activation of the caspase cascades has a dominant role in developing the muscle cell’s injuries. **Abbreviations**: Cyt-C, cytochrome-C; ERK, extracellular signal-regulated kinases; Fas, apoptosis antigen 1 (APO-1 or APT); IL-1R, interleukin-1 receptor; IL-1β, interleukin-1β; MAPK, mitogen-activated protein kinase; NFκB, nuclear factor kappa-light-chain enhancer of activated B cells; ROS, reactive oxygen spices; TNF-R, tumor necrosis factor receptor; TNF-α, tumor necrosis factor-α; TRAIL, TNF-related apoptosis-inducing ligand; TRAIL-R, TNF-related apoptosis-inducing receptor. Figure created with BioRender.com.

It is reported that oxidative stresses, which interfere by increasing intracellular levels of reactive oxygen and nitrogen species (ROS and RNS), are the primary disruptive factors in the ischemia pathogenesis path (Schröder et al., 2012; Bagheri et al., 2016). Meanwhile, all limb tissue cell types, including endothelial cells, skeletal myocytes, fibroblasts, and peripheral neurons, have been shown to present identical pathogenic responses to intracellular ROS and RNS generation (Steiling et al., 1999; Wang et al., 2015; Cheng et al., 2016). Activation of reactive species can directly cause cell damage through DNA sequence alteration, gene amplification, and expression of some proto-oncogenes and tumor suppressor genes (Bagheri et al., 2016; Qiu et al., 2019). In most cellular populations, mitochondria organelles are the main targets of ROS-mediated signaling in the path of HLI pathogenesis. In more detail, production of ROS is directly controlled by the ischemic cell’s mitochondria electron transport chain (ETC) overactivity (Indo et al., 2007). Moreover, ROS directly switches cytoplasmic Ca^2+^ accumulation. This mechanism leads to interference of the injured cell’s mitochondrial permeability transition pore (mPTP) opening and affects the mitochondrial membrane potential (Batandier et al., 2004). This mitochondrial mechanism immediately stimulates apoptosis cell death in the ischemic tissues by releasing cytochrome-c protein into the injured cell’s cytoplasm, which, in turn, progresses the caspase cell death cascade (Figure 1). Aside from the cytochrome-c accumulation, some other harmful function cascades are caused by the ROS/mitochondia interaction with the injured cells. The cytoplasmic expression of apoptotic factors like B-cell lymphoma 2 (BCLl-2), BCL-2-associated X protein (BAX), BCL-2-associated death promoter (Bad), and glycogen synthase kinase 3β (GSK-3β) are the other main factors involved in this path (Perrelli et al., 2011) (Figure 1).

In contrast with the apoptotic mechanisms, several protective pathways can be initiated by the stressed mitochondria in response to ROS (Stein et al., 2017). The protein kinase C (PKC) signaling pathway is one of the main types of these protective mechanisms, which also plays multiple roles in the development and hemostasis of organs (Stein et al., 2017; Khodayari et al., 2019). In addition to the mitochondrial electron transport chain (ETC), the PKC and nicotinamide adenine dinucleotide phosphate (NADPH) oxidases, other main cell protective factors could be broadly released from different origins, including the xanthine oxidase, cyclooxygenase, and lipoxygenase (Leslie et al., 2004). In this regard, the generated ROS can directly target the PKC release into the injured cell cytoplasm through stimulation of the mitochondrial function. From another path, the activated PKC directly generates ROS production through NADPH phosphorylation (Lee et al., 2004). The activated NADPH improves cells’ survival and function inside the stressful environment through activation of protective signaling pathways, including the apoptosis signal-regulating kinase-1 (ASK1) and AKT pathways (Cosentino-Gomes et al., 2012) (Figure 1).

In addition to PKC, the mitogen-activated protein kinases (MAPKs), stress-activated protein kinases (SAPKs), phosphoinositide 3-kinase (PI3K), and ataxia-telangiectasia mutated (ATM) pathways can also serve as cell protective mechanisms which become activated as a result of ROS activity (Cosentino-Gomes et al., 2012). It has been identified that progression of both MAPK and SAPK activation of Janus kinase (JAK)/signal transducers and activators of transcription (STAT) signaling can protect the injured cells from the disruptive impacts of oxidative stress. In this described cascade, activated PIK3 can significantly protect injured cells as well as distant cells by promoting 3 phosphoinositide-dependent protein kinase-1/Akt (PDK1/Akt) signaling (Cosentino-Gomes et al., 2012). The aforementioned pieces of evidence intensively prove this hypothesis, which states that ROS can directly act as a “double-edged sword” in the HLI pathogenesis process (Figure 1).

In addition to the described mitochondrial-associated mechanisms, non-mitochondrial ROS-mediated cell death plays a major role in limb tissue oxidative stress. In this process, generation and activation types of well-known inflammatory network elements including tumor necrosis factor-α (TNF-α) and its downstream targets have a central role in evolving the cellular death and limb tissue degeneration (Seekamp et al., 1993). It seems that this referred pro-inflammatory cytokine stimulates the same pathological responses for all organs and tissues, as well as the HLI (Khodayari et al., 2019). It has been shown that in following the limb tissue ischemia, the TNF-α production is directly initiated by overexpression of some adhesion molecules from ischemic tissue’s vascular endothelial cells (Simon et al., 2018). According to contemporary observations, TNF-α is the initial and primary pro-inflammatory paracrine factor that directly switches the cytokine changes in the ischemic microenvironment (Heath et al., 2018). TNF-α, through the stimulation of leukocytes, initiates the inflammation process and recruitment of the inflammatory immune cells into the ischemic injured limb tissue. Subsequently, as a result of this mechanism, the expression of other types of pro-inflammatory cytokines, including interleukin-1β (IL-1β), interleukin-6 (IL-6), and interleukin-8 (IL-8), occurs in infarcted tissues (Khodayari et al., 2019). It should be noted that this complex and stressful environment is considered the main reason for different inflammatory/degenerative disorders (Figure 1).

Furthermore, following the TNF-α associated pathogenicity, stimulation of the TNF-α receptors (TNF-Rs) as one of the main inflammatory cell death ligands can play the pivotal role in tissue degeneration (Sedger and McDermott, 2014). Promoting nuclear factor-kappa B (NF-κB) cascade, following TNF-R activation, can induce the cellular death process (apoptosis and necroptosis) into the injured cells (Kim et al., 2011). Like TNF-α, IL-1β-induced NF-κB activation is another pathogenic factor that occurs after the HLI process (Khodayari et al., 2019). The expressed NF-κB, through the activation of other pro-apoptotic factors including the inducible nitric oxide synthase (iNOS), BH3 interacting domain death agonist (Bid), and c-Jun N-terminal kinases/P38 (JNK/P38), can conduct the limb cells toward apoptosis (Cnop et al., 2005). Finally, it should be mentioned that all of the aforementioned adverse mechanisms are just a meager part of the pathogenic and progressive process that is activated in the ischemic limb tissue microenvironment.

## Stem Cell Therapy Approaches for Limb Ischemia in Practice

In the past decade, stem cell-based therapy approaches have become extremely useful facilitators for the regulation of different types of human disorders, including acute and chronic limb ischemia (Zafarghandi et al., 2010; Mardanpour et al., 2019). From a translational and practical view, the performance of some multipotent stem/progenitor cells, including the BM-MNCs, CD34^+^ MNCs, MSCs, and ESPCs, has been approved and deeply studied in different clinical trials (Supplementary Table S1). Furthermore, the therapeutic effects of EnSCs and NSCs are investigated in some published and ongoing clinical trials on limb lesions (Supplementary Table S1). Generally, the therapeutic benefits from these stem cells can be used in all phases of limb ischemia (Jaluvka et al., 2020). However, types of signs include patient survival rate lower than 6 months, history of malignancy, chronic renal disorders on dialysis therapy, or ALI cases with an advanced inflammatory reaction considers as the contraindication of the LI stem cell therapy (Jaluvka et al., 2020).

In this section, we will briefly review the biological function and regenerative outcomes of the aforementioned stem cells based on the latest clinical observations. Finally, at the end of this section, we will present the EnSCs as an “all-in-one” therapeutic approach for limb regeneration, based on our findings.

### Bone Marrow-Derived Mononuclear Cells

Generally, the BM-MNCs, as a heterogeneous cell population, are carefully characterized through the positive expression of CD34, CD45, CD133, and stromal precursor antigen-1 (STRO1) markers from bone marrow aspirated cells (Liang T. W. et al., 2016). It has been reported that the injected BM-MNCs, through the release of various anti-inflammatory cytokines and growth factors (Table 1), could regulate the ischemic region’s pathogenic mechanisms. In addition, the vasculogenic, myogenic, and neurogenic differentiation potentials of these cells have been clearly recognized by numerous investigations (Table 1). According to these complementary features, the BM-MNCs are considered to be a major population in the target regeneration. However, some main drawbacks, such as the high possibility of contamination leading to a long time to culture and mainly decreased cell volume and regenerative capacity due to the increasing age of patients, can affect the use of the BM-MNCs for limb regeneration (Laurence et al., 2015).

So far, different studies have been conducted in order to evaluate the benefits of BM-MNC transplantation as a means of managing limb ischemic injuries (Supplementary Table S1). From 2008 to 2011, we launched two separate clinical trials (phases I/II randomized control trials) for the autologous BM-MNC therapy of patients with CLI (ClinicalTrials.gov identifiers: NCT00677404 and NCT01480414). In those continued clinical observations, we focused on the most pressing questions, including 1) is autologous transplantation of the BM-MNCs safe in patients with CLI, 2) how much simultaneous injection of granulocyte-colony stimulation factor (G-CSF) with the transplantation of BM-MNCs would increase the efficacy of cell therapy in CLI patients, 3) can interval implantation of the BM-MNCs improve the treatment efficacy, and 4) can the BM-MNCs interval injection increase collateral vessel formation in patients with limb ischemia? Following these trials, we stated that the administration of BM-MNCs is a safe and feasible approach for the treatment of patients. Our evaluations additionally showed that the intervention hand was able to improve some patients’ clinical indexes, including pain-free walking distance and Wagner stage, besides reduction in ulcer size (Zafarghandi et al., 2010; Molavi et al., 2016).

In a successful study, Tateishi-Yuyama et al. (2002) published an article titled “therapeutic angiogenesis for patients with limb ischemia by autologous transplantation of bone-marrow cells.” Their clinical trial has shown that direct intramuscular injection of CD34^+^ BM-MNCs not only is able to induce an angiogenic response but can also significantly improve the patient’s limb function and ankle-brachial pressure index (ABI) 6 months after cell therapy (Pignon et al., 2017). Similar to the aforementioned study, current clinical observations are also displaying an identical therapeutic impact that occurred from the BM-MNCs administration on the limb ischemia cases. Pignon et al. (2017), through intramuscular injection of the CD34^+^ BM-MNCs on patients with CMI, observed a suitable and remarkable therapeutic response within 6 months after cell therapy. Finally, they concluded that “BMSC therapy reduced the risk of major amputations in patients presenting with nonrevascularizable CLI” (Pignon et al., 2017).

Similarly, autologous bone marrow mononuclear cell (ABMNC) therapy is another approach that improves measures of limb perfusion, rest pain, wound healing, and amputation-free survival at 1 year in patients with CLI. As an example, Tateishi-Yuyama et al., in a retrospective study, evaluated the incidence of cardiac, malignant, and other medical events relevant to the safety of cell therapy. They found that none of the patients developed tumorigenesis or clinically significant retinopathy. Mainly, they found that in arms treated with ABMNC, the rates of amputation-free survival, major amputation, and major adverse limb events were significantly higher than in the control group (Tateishi-Yuyama et al., 2002).

**FIGURE 2:**
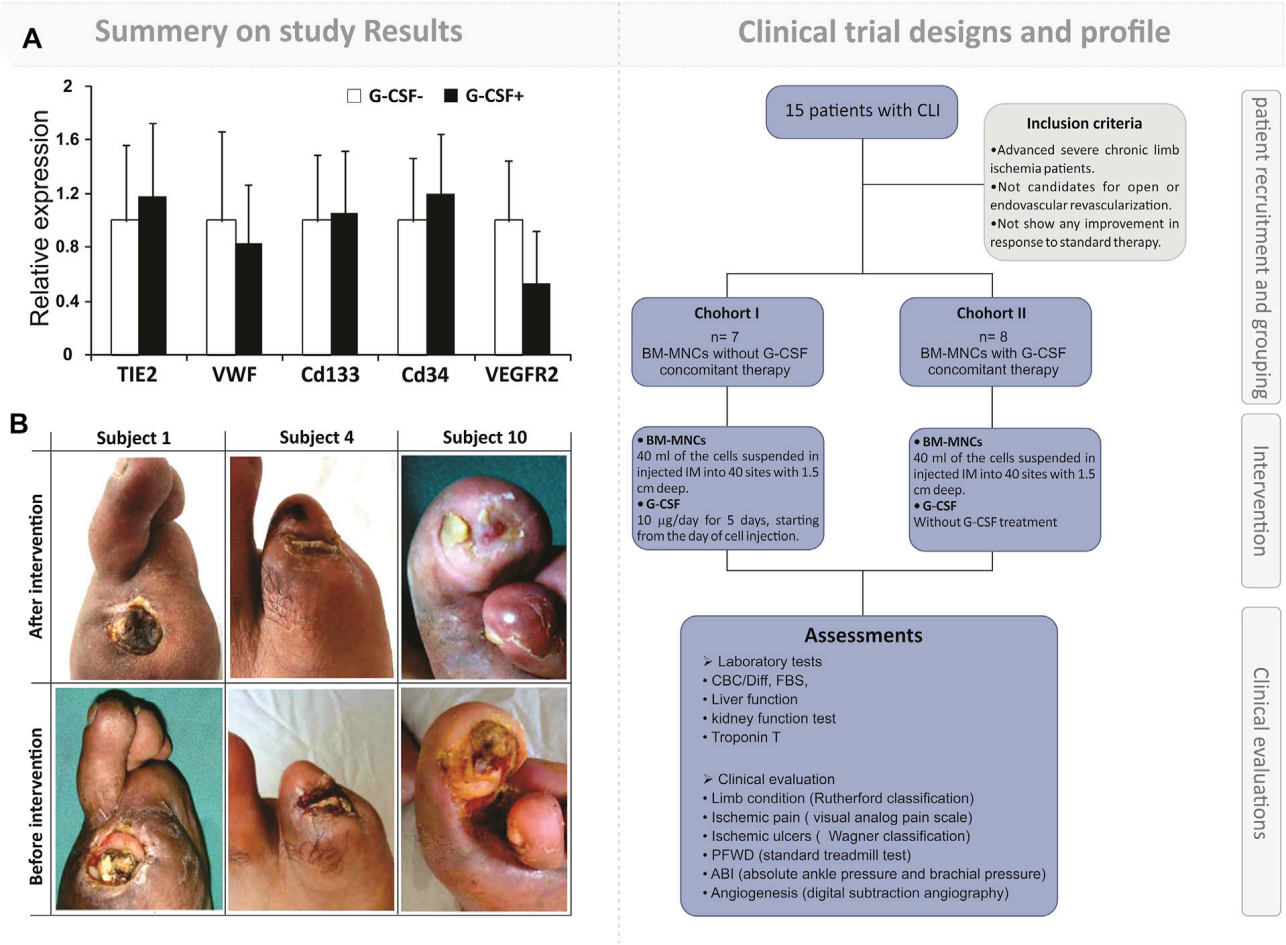
Outcome of simultaneous autologous BM-MNC and G-CSF administration to patients with limb ischemia. **First part**: a brief representation of the flowchart for the clinical trial design and assessments. The study was a phase I randomized controlled trial on fifteen patients with severe CLI. The intervention’s safety and efficacy were evaluated by laboratory analysis, clinical evaluation, and imaging. **Second part**: **(A)** qRT-PCR analysis of the expression of angiogenesis markers in two arms, BM-MNC alone vs. BM-MNC plus G-CSF. **(B)** Improvement in limb salvage after autologous BM-MNC therapy. Zafarghandi, Mohammad Reza, et al. Cytotherapy 12.6 (2010): 783–791.

Although in recent years, scientists have announced some remarkable therapeutic methods for regenerating the limb ischemia cases based on the administration of BM-MNCs, various gaps and problems have already existed in this path (Liang T. W. et al., 2016). It seems that there was a correlation between the reputation and numbers of the implanted BM-MNCs and regeneration outputs in the IL cases. Hence, our observation in 2016 has been compared with the therapeutic output that resulted from a single and repeated (4 step) intramuscular injection of 1–2×10^5^ BMDC/kg on CLI patients (Figure 2). The results of this study revealed a significant improvement in the repeated treated cases “ABI, visual analog scale, pain-free walking distance, and Wagner stage as well as a reduction in ulcer size” in comparison with the single-injected CLI patients (Zafarghandi et al., 2010). However, it should be noted that the BM-MNCs are also showing satisfactory efficacy in ischemic limb regeneration (Supplementary Table S1). The achievement, compared to the other heterogeneous and high-potent stem cell populations with a suitable regenerative potential, can be presented as an effective approach along the lines of managing limb ischemia regeneration.

### Mesenchymal Stem Cells

Based on several hypotheses, presenting a large level of MSCs into the BM-MNC population introduces a key method in order to manage an effective therapeutic strategy for the regeneration of ischemic disorders (Wang et al., 2018; Khodayari et al., 2019; Mardanpour et al., 2019). Moreover, among the BM-MNC cellular population, the MSCs have shown more effectiveness for improving the ischemic limb tissues based on the particular observations in comparison with the other stem cells (Mafi et al., 2011; Gupta et al., 2013). In addition to the bone marrow, the MSCs can be isolated from different origins like fatty pads, synovium, and cord blood (Khodayari et al., 2019). Secreting a high level of paracrine factors, increased proliferation and differentiation potential, ability to utilize as an allogeneic source, elevated resistance to inflammation as well as lower teratogenic/carcinogenic potential considers the main advantages of the MSCs for the regenerating targets (Khodayari et al., 2019; Khalighfard et al., 2021). In contrast, the requirement to use invasive methods for getting a biopsy sample, the dependence of the cells’ proliferative and differentiation capacity with age, and the need to use various markers for producing a homogeneous population are the cells’ main disadvantages (Matsiko et al., 2013). Phenotypically, in humans, the BMSCs are a fibroblast-like cell population that is generally distinguished by the positive expression of STOR1, CD73, CD90, and CD105, besides the negative expression of CD11b, CD14, and CD34. Also, as negatively marked, CD79a and HLA-DR can be noticed in the MSCs (Khodayari et al., 2019). In addition to the bone marrow, human MSCs can be isolated from different niches, including the adipose tissue (Yañez et al., 2006), umbilical cord blood (Hong et al., 2005), and dental tissue (Xu et al., 2020).

There are several successful clinical methods for evaluating the regenerative impacts of MSCs on limb ischemia disorders. Gupta et al. (2013) have shown that intramuscularly injected BM-MSCs not only were safe but also decreased the CLI patient’s limb pain and improved their ABI and ankle brachial pressure index (ABPI). Also, they have shown that the numbers of the administrated CM-MSCs are able to positively change the cell therapy output in CLI cases (Gupta et al., 2013). Das et al. (2013), *via* an intra-arterial allogeneic MSC infusion into CLI patients, could recognize a safe and effective response in their study. Based on their reports, not only did the MSCs treated group show significant pain relief, but it also greatly improved the other clinical indexes, including the visual analog scale (VAS), ABPI, and transcutaneous oxygen pressure (TcPO_2_) (Das et al., 2013). Other clinical observations have only shown an unstable and limited regeneration response from the MSC subtype implant. Adipose-derived stromal/stem cells (ADSCs) are introduced as a mesenchymal stem-like population with the same molecular phenotype as MSCs (Zannettino et al., 2008). Moreover, Bura et al. (2014), through their phase I clinical trial on nonrevascularizable CLI patients, observed that even though intramuscularly injected ADSCs in the ischemic tissue were feasible and safe, the case’s clinical index was not sufficiently improved after the cell therapy (Bura et al., 2014). This variation and huge difference between the MSCs and the MSC-like regenerative responses may be dependent on the variations of the cell’s niches, paracrine/autocrine activity, and differentiation potential.

Generally, stimulating the angiogenic mechanisms in the MSCs seems to be a powerful approach for improving the neovascularization response from the implanted MSCs (Yu and Dardik, 2018; Goodarzi et al., 2020; Khalighfard et al., 2021). The upregulation of hypoxia-inducible transcription factor-1α (HIF-1α), as a chief angiogenic regulator, could be used as a feasible and effective procedure that could achieve this result. During this process, as the main downstream target of HIF-1α, activation of remarkable angiogenic signaling pathways including the vascular endothelial growth factor (VEGF), epidermal growth factor (EGF), transforming growth factor-β (TGF-β), and small mothers against decapentaplegic 3/4 (SMAD 3/4) can greatly improve the human MSCs’ endothelial differentiation in the ischemic limb microenvironment (Huang et al., 2016). This hypothesis has been successfully proven by Wang et al., 2015), following their preclinical observations (Huang et al., 2016). Through intramuscular injection of 1×10^6^ HIF-1α upregulated human MSCs, they recorded a meaningful angiogenic response and observed the injured limb tissue regenerate early after the cell transplantation (Howangyin et al., 2014).

### CD34-Positive Mononuclear Cells

As a population of MNCs, the CD34^+^ mononuclear cells present a non-hematopoietic stem cell with an innate potential for tissue regeneration (Johnson et al., 2020). Different origins such as umbilical cord blood, bone marrow, and peripheral blood are utilized for the isolation of high-potent CD34^+^ MNCs to be used as a therapeutic factor (Bender et al., 1994; Johnson et al., 2020). In this case, BM and PB-CD34^+^ MNCs are the most commonly trialed cells for the treatment of ischemic limbs in practice (Supplementary Table S1). Generally, these cells can be isolated by positive expression of CD34 markers along with negative expression of CD14 and CD45 transmembrane proteins (Table 1). Different experimental observations have concluded that the PB-CD34^+^ MNCs could directly generate types of cellular lineages like myocytes (Avitabile et al., 2011), endothelial cells (Krenning et al., 2009), and neural cell lineages (Reali et al., 2006) that are vital for injured limb regeneration. In addition to their differentiation potential, the release of different therapeutic factors, including 1) cytokines and immune suppressors, 2) growth factors and morphogens, 3) chemokines and surface regulatory markers, and 4) extracellular vesicles, can be used as other therapeutic effects resulting from the PB-CD34^+^ MNCs implantation. A list of the main PB-CD34^+^ MNCs secretomes is categorized in Table 1.

In order to increase the mobilization of the CD34^+^ MNCs into circulation, the most common approach is to administer G-CSF days before cell isolation (Kawamoto et al., 2009). It has been shown that the pharmacological effects of G-CSF on BM stem cell populations are directly promoted *via* activation of signal transducer and activator of transcription 3 (STAT3) and rat sarcoma (RAS)/rapidly accelerated fibrosarcoma (RAF) signaling (Kamezaki et al., 2005). These pathways serve as types of central mechanisms which promote cellular proliferation and migration from bone marrow to transmission. So far, different experimental and clinical trials have evaluated the safety and effectiveness of the PB-CD34^+^ MNCs in the various types of LIs (Kawamoto et al., 2009). Wahid et al. (2018) wrote in their meta-analysis for evaluating the “efficacy and safety of autologous cell-based therapy in patients with no-option critical limb ischemia” that the MNCs, mainly bone marrow-driven cells, are safe stem cells with more therapeutic potential for the regeneration of CLI (Wahid et al., 2018). In one of the earliest clinical trials, Kinoshita et al. (2012) examined the long-term clinical outcome of IM G-CSF-mobilized CD34 MNC injection in patients with CLI (Kinoshita et al., 2012). Their observation demonstrated that the IM injection of PB-CD34^+^ MNCs had favorable and long-term clinical benefits in these types of patients. As a result, they have recorded an improvement in brachial pressure index and transcutaneous partial oxygen pressure up to 6 months after cell therapy (Kinoshita et al., 2012).

It seems that in addition to using a sufficient number of a viable and homogenous population of CD34^+^ MNCs, the characteristics of subjects can be a determining issue for performing efficient cell therapy in LI patients. Pan et al. (2019), phase I/II clinical trial, showed that the total number of transplanted cells, patient’s age, blood fibrinogen, arterial occlusion level, and TcPO2 directly affected the outcome of PB-CD34^+^ MNCs therapeutic angiogenesis for no-option CLI cases (Pan et al., 2019). Moreover, the different observations identified suitable outcomes of the PB-CD34^+^ MNC therapy in CLI patients with atherosclerotic peripheral arterial disease and Buerger’s disease (Supplementary Table S1).

### Endothelial Progenitor Cells

Endothelial progenitor cells (EPCs) have been utilized to generate various cell types that have a key role in endothelial lining regeneration and neovascularization (Esquiva et al., 2018). Generally, EPCs are generated in the bone marrow environment by the CD34^+^/CD45^+^/CD133^+^ hematopoietic stem cells (HSCs) and non-hematopoietic bone marrow cells, including the MSCs (Urbich and Dimmeler, 2004). EPCs are collected within the positive expression of CD133, CD34, and vascular endothelial growth factor receptor-2 (VEGFR2) directly from the bone marrow or circulating blood cells (Urbich and Dimmeler, 2004; Friedrich et al., 2006). It has been reported that in addition to neovascularization into the injured tissues, utilized EPCs can modulate the stressful organ’s microenvironment through expression of diverse types of angiogenic growth factors like VEGF, hepatocyte growth factor (HGF), and insulin-like growth factor 1 (IGF-1) (Abe et al., 2013).

In the past, some clinical observations have considered the use of EPC’s regenerative potential for patients with limb ischemia. As a primer clinical observation, Kudo et al. (2003) stated that intramuscular injection of the autologous CD34^+^ EPC can be a powerful method for regenerating ischemic limb tissues. The results they presented after EPC therapy read “transcutaneous oxygen pressure in the foot increased and clinical symptoms improved.” Newly visible collateral blood vessels were directly documented by angiography (Kudo et al., 2003). The important physiological function of the EPCs on the limb ischemia patient’s therapeutic response rate has already been discovered. Based on this hypothesis, the lower neovascularization rate into the limb ischemia patients who are showing a minor therapeutic response to the treatments may be related to their EPC defect. Flow cytometry and EPC quantitative molecular analysis on ischemic limbs patients with a moderate risk of neovascularization, showed that not only the number of their circulating EPCs was lower but also the proliferation and differentiation potential of their collective EPCs was not as high as it was in the control cases (Yamamoto et al., 2004).

It has been found that stimulation of the EPCs with pro-inflammatory cytokines, like IL-1β, IL-3, and TNF-α, significantly decreases the proliferation and angiogenic potential of the cell. Accordingly, to perform an effective cell therapy on the ischemic limbs, based on using the EPCs, production of them at a large level are required (Peplow, 2014). We believe that this is the main disadvantage that leads to the reduction of this regenerative method’s cost-effectiveness.

### Neural Stem Cells

Peripheral neuropathy associated with limb ischemia is one of the prevalent pathogenesis that is commonly observed in patients with vascular disease (Uccioli et al., 2018). “Chronic pain, foot ulcers, foot infections, and amputations” manifest as chief complications caused by peripheral neuropathy (Hicks and Selvin, 2019). Nowadays, using stem cell technology for regeneration of the damaged peripheral neural system (PNS) presents an applicable, safe, and efficient method (Sullivan et al., 2016). This discovery of NSCs was an impactful development for the sake of eventually achieving a regenerative method for the injured PNS. The NSCs are a population of undifferentiated cells that naturally persist in the mammalian ventricular-subventricular zone (V-SVZ) and the subgranular zone (SGZ) (Fuentealba et al., 2012). In humans, this cellular population is noted by positive expression of CD184, CD24, nestin, fibroblast growth factor receptor (FGF-R), glial fibrillary acidic protein (GFAP), SRY-box transcription factors 1/2 (SOX1/2), forkhead box O3 (FOXO-3), and orphan Nuclear Receptor TLX, as well as negatively expressed CD271 and CD44 markers (Table 1). Different observations proved the neural linage differentiation potential of NSCs in both *in vitro* and *in vivo* conditions (Harbom et al., 2018). In addition to the release of various paracrine and growth factors, these stem cells can also express types of chemokines and cell migration receptors (Table 1).

The large-scale production of NSCs for managing an effective cell therapy in patients with PNS disorders presents a principal challenge. Extreme risks of taking a biopsy from a patient’s SVZ and SGZ, the presence of small numbers of NSCs in the received tissue, and finally, the poor proliferative potential of the hNSCs primary cultures are some of the limitations presented in this approach (Kaneko et al., 2011). However, like the EPCs, resistance to the ischemic organ inflammatory responses along with the high cost of the NSCs’ isolation, culture, and purification prevents the widespread use of these cells in the practice (Pluchino et al., 2020). Nowadays, using gene transfection technology to generate off-the-shelf NSCs lines serves as a feasible and satisfactory approach. As a pioneer product, the human NSC-line CTX (CTX0E03) created with the MYCL proto-oncogene (c-myc)-ERTAM transfection performs as an off-the-shelf allogeneic stem cell for the CNS and PNS regeneration targets, in practice (Yoon et al., 2020). Results of the Muir et al. (2020) clinical trial on twenty-three patients with stroke confirmed that intracerebral implantation of the CTX cells was not only a feasible approach for nerve regeneration but also had a positive outcome on the subject’s motor recovery up to 12 months after implantation (Muir et al., 2020). A phase I, randomized, controlled trial has been launched in order to investigate the safety of CTX0E03 cell line implantation in patients with “insulinized diabetic patients type 2 with CLI in the lower limb” (ClinicalTrials.gov Identifier: NCT02287974). Although results have yet to be posted from this ongoing study, evidence raised from Muir et al. (2020) study may suggest that using these universal cell lines could be a safe and efficient cell therapy method for patients with LI.

## Endometrial-Derived Stem Cells: All-In-One for Limb Regeneration

**FIGURE 3:**
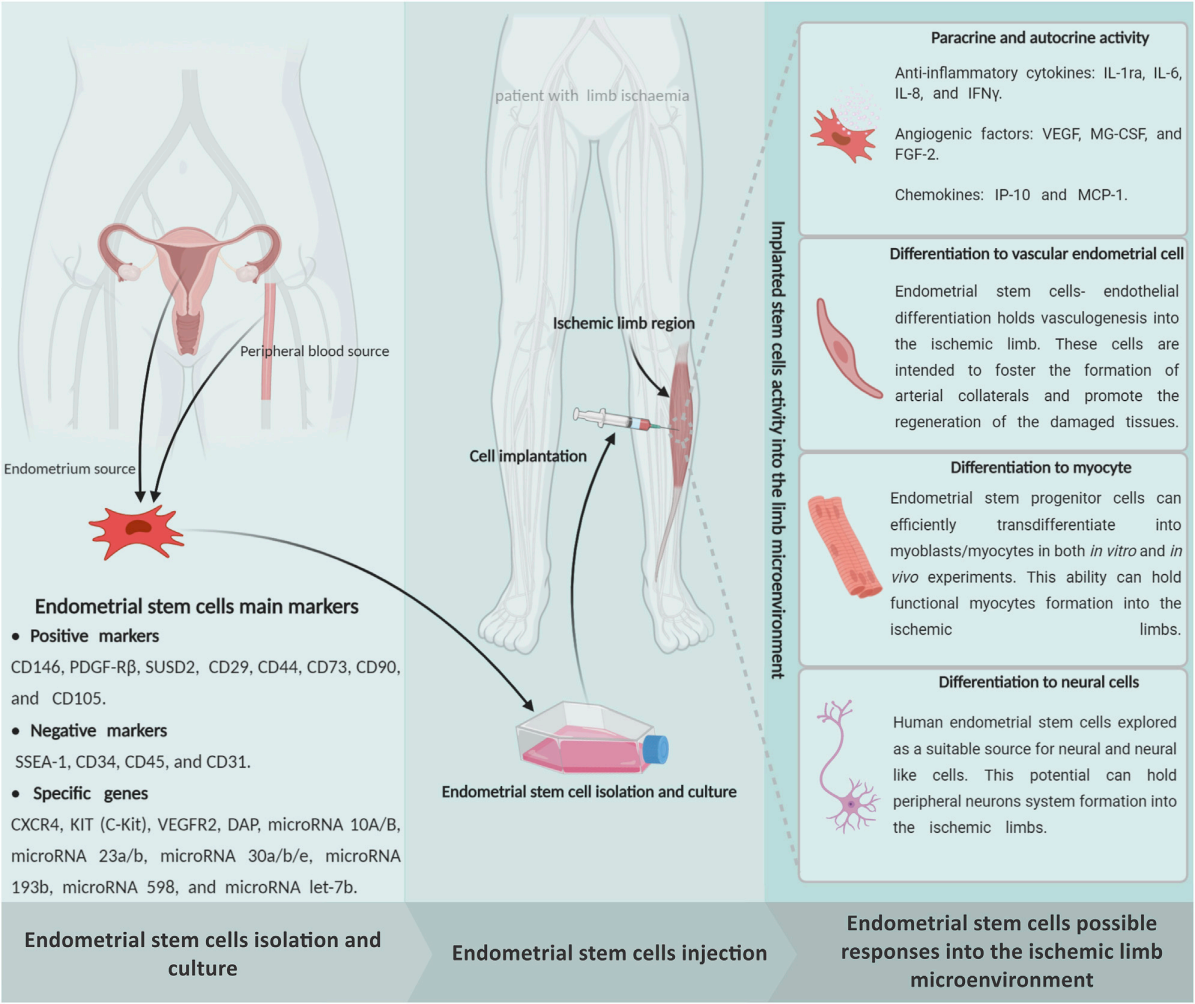
Schematic representation of the endometrial stem cells possible impact on the limb ischemia regeneration subjects. The endometrial stem cells can be directly isolated from an endometrium biopsy through exposure of endometrial, mesenchymal, and endothelial specific markers. It seems that implantation of the *in vitro* expanded endometrial stem cells into the ischemic regions is able to protect the damaged cells from cellular death through a powerful paracrine/autocrine activity. In addition to, as a multipotent stem cell, the endometrial stem cells can generate the main limb linage cells, which may promote effective regeneration in the ischemic limbs. Figure created with BioRender.com.

As we explained, different stem cells have been utilized in many preclinical and clinical experiments over the past 5 decades due to their extraordinary capacity for differentiation into multiple lineages, immunomodulation, and angiogenic properties (Supplementary Table S1). During the past few years, our team’s extensive observations into EnSCs have displayed this stem cell’s potential for regenerating ischemic organs as well as limb tissue. The presence of stem cells in the endometrium was first speculated upon by Prianishnikov (1978) (Prianishnikov, 1978). Chan et al. (2004) also confirmed the presence of clonogenic stem cell populations in the endometrium tissue (Chan et al., 2004). We already proved that this mentioned regenerative capacity in the endometrium tissue is due to the presence of EnSCs that were demonstrated to be immuno-privileged in comparison with other cell types (Shamosi et al., 2015), shedding new light on cell-based therapies and rendering these cells a promising resource in limb tissue regeneration (Figure 3). The human endometrium is a highly regenerative tissue due to the fact that a complete regeneration of the uterus can be achieved after almost total resection of the endometrium, with the renovated endometrium still maintaining its ability to support gestation (Murphy et al., 2008). Monthly cyclical endometrial growth, differentiation, and regression are governed by a fine-tuned interplay between ovarian sex steroid hormones and numerous cell types. It has been shown that estrogen replacement therapy can regenerate atrophied endometrium (Evans et al., 2016). The endometrial tissue is composed of epithelial stem cells (0.22%), mesenchymal stem cells (1.25%), and side population stem cells (Sasson and Taylor, 2008). During menstruation, all of these cells could be found as menstrual stem cells or endometrial regenerative cells (ERC) (Verdi et al., 2014). Like the other sources, endometrial MSCs have recently emerged as a promising alternative source of therapeutic MSCs due to their unique ability to undergo smooth muscle differentiation (Rink et al., 2017), angiogenic (Cabezas et al., 2018), adipogenic (Ye and Yuan, 2007) and robust immunomodulation properties (Cortés-Araya et al., 2018). *In vitro* analysis reported that the EnSCs positively express stage-specific embryonic antigen-4) SSEA-4 (and octamer-binding transcription factor 4 (OCT4) markers and decrease OCT4 expression with increasing passages that confirm its stemness (Piccinato et al., 2015). The EnSCs also expressed high levels of CD73, CD90, CD105, and CD166, but they lacked CD14, CD34, and CD45 expression, which are very similar to the minimum characteristics of MSCs defined by the committee of the International Society for Cellular Therapy (Piccirillo et al., 2013). The EnSCs also have a high proliferation, multidirectional, and differentiation capacity that is indicative of the potential for tissue regeneration (Ai and Ebrahimi, 2010).

As an advantage, the EnSCs have been shown to have the same immunophenotype as the MSCs. These cells express MHC class I (HLA-ABC) but not MHC II (HLA-DR). They have the capability to modulate immune system functions, reduce proinflammatory cytokines including the TNF-α and interferon gamma (IFN-γ), and have been shown to inhibit mixed lymphocyte reactions (MLR) in different experiments (Cheng et al., 2017). As a result, EnSCs have been shown to have low immunogenicity and various *in vivo* experiments have been performed without the occurrence of immune rejection (Giudice, 1994). Moreover, as well as the MSCs, the EnSCs can directly synthesize and discharge several types of paracrine factors, including the TGFα, EGF, and IGF-I (Figure 3). IGF-I, TGFα, epidermal growth factor receptor (EGFR), basic fibroblast growth factor (bFGF), and platelet-derived growth factor-β (PDGFβ) receptor, which are expressed in epithelial cells, are upregulated during expeditious growth in the proliferative stage of stem cells. Leukemia inhibitory factor (LIF), hepatocyte growth factor (HGF), and stem cell factor (SCF) have an essential contribution to endometrial regeneration (Chegini et al., 1992). All of the EnSC paracrine factors have an essential role in ischemic limb regeneration.

The novel hypothesis considers angiogenesis as the most remarkable regenerative approach, along with stem cell therapy for limb ischemia patients. We strongly believe, due to the high similarity between the EnSCs and the MSCs, that evaluating the MSCs’ angiogenic responses in the ischemic tissue’s microenvironment can be an accurate model to study for the EnSCs’ angiogenic behaviors (Ai and Ebrahimi, 2010). The MSCs exhibit angiogenic properties by not only paracrine signaling but also by regulating this response through direct cellular involvement. An array of angiogenic factors secreted by MSCs, for example, VEGF, SDF1, FGF2, HGF, angiopoietin-1 (ANG1), and monocyte chemoattractant protein-1 (MCP-1), act as key growth factors for primary vessel formation and its stabilization (Tao et al., 2016). Moreover, *in vitro* and *in vivo* studies showed that the microvesicles (>200 μm) and exosomes (∼50–200 μm) secreted by MSCs transport proangiogenic growth factors and miRNA (Phinney and Pittenger, 2017). A proteomic analysis showed that enhanced therapeutic angiogenesis, resulting from the secretion of MSC-derived exosomal proteins (PDGF, FGF, EGF, NF-κB pathway-affiliated proteins, etc.) into the ischemic region, has been observed after MSC therapy (Anderson et al., 2016). X.Liang T. W. et al., 2016) demonstrated that adipose MSC-derived exosomes transfer miR-125a to endothelial cells and promote angiogenesis (Liang X. et al., 2016). Also, Gong et al. (2017) have investigated whether the proangiogenic exosomal miRNA (miR-424, miR-30c, miR-30b, let-7f, etc.), derived from MSCs, can be transferred into endothelial cells (Gong et al., 2017).

Many investigations have shown that BM-MSCs have a strong capability for angiogenesis induction (Kinnaird et al., 2004). Clearly, the EMSCs and BM-MSCs share some common cell surface markers like CD90 and CD105, while lacking CD45 and CD34 (Keating, 2012). A series of experiments have been performed *in vitro* and *in vivo* to assess the proof of an angiogenic concept on endometrial derived stem cells, and they showed that EnSCs could stimulate angiogenesis (Murphy et al., 2008). Results from our team’s studies have shown that human EnSCs could proliferate and sprout new blood vessels in 3D fibrin matrix supported culture (Esfandiari et al., 2007a; Esfandiari et al., 2007b; Esfandiari et al., 2008). Similarly, in 2013, we were able to prove the EnSCs’ direct potential to generate CD34^+^ endothelial cells (Ai et al., 2013). In addition, according to data from one of our *in vitro* experiments, human EnSCs have shown that they can be differentiated into endothelial-like cells in the presence of FGF-2 and VEGF on a nanofibrous scaffold (Shamosi et al., 2017). Following this discovery, in 2004, we demonstrated the vascular network formation and the angiogenic potential of EnSCs through an *in vitro* examination (Esfandiari et al., 2008). It is important to note that EnSCs are found to be clonogenic mesenchymal-like cells (Gargett et al., 2009), which express pericyte markers and are localized in the perivascular space of endometrial small vessels (Berger et al., 2005). These cells play a central role in the formation of endometrial stromal vascular tissue and vascularization through the secretion of pro-angiogenic and growth-supporting factors. Canosa et al., 2017) concluded that the EnSCs could support the endothelial cell’s ability to differentiate by taking information from the endothelium and new blood vessels (Canosa et al., 2017). Due to these findings, we believe that the EnSCs can be considered as an impactful stem cell for managing effective regenerative angiogenesis in the injured limb tissue microenvironment (Figure 3).

Aside from angiogenesis, reconstructing the failed myocytes into the ischemic limb area is introduced as another target along with limb tissue regeneration. In this regard, our team’s remarks claimed that the EnSCs seem to be a suitable cellular source for regulating myogenesis in addition to the other well-trialed sources like the MSCs. Various experimental study showed that MSCs could differentiate into skeletal muscle cells and could be a feet source for repair (Gang et al., 2004; Sassoli et al., 2014). Gang et al. (2004) isolated MSCs from umbilical cord blood and induced them to differentiate into skeletal muscle cells. They recommended this stem cell phenotype as a useful tool for muscle-related tissue engineering (Gang et al., 2004). A few years later, Toyoda et al. (2007) described the EnSCs derived from menstrual blood have the remarkable myogenic potential that could allow “rescue” dystrophied myocytes in the MDX model through cell fusion and transdifferentiation (Toyoda et al., 2007). In their work, Cui et al. (2007) showed that endometrial progenitors and menstrual blood-derived mesenchymal stem cells could efficiently transdifferentiate into myoblasts/myocytes, both in *in vitro* and *in vivo* experiments. They expected that the EnSCs could be a major advancement toward cell-based therapies for chronic muscular diseases and muscle injury (Cui et al., 2007). However, based on our investigations, we have a more transcendent view of EnSCs as a powerful tool for myocyte regeneration. In 2009, we carefully hypothesized that EnSCs could be a more suitable option for muscle regeneration cell therapy, based on the properties of EnSCs, including easy accessibility, easy purification, clonogenicity, and maintenance of normal karyotyping in extended passages (Cui et al., 2007). Also in 2014, one of our lab’s investigations showed that the EnSCs could serve as a potent myogenic cell source, having a superior differentiation capacity over that of BM-MSCs that was fully supported by Real-Time polymerase chain reaction (PCR) and immunocytochemistry to detect myogenic markers and quantitative-PCR for the upregulation of desmin, myoblast determination protein 1 (MyoD), and troponin T transcripts (Faghihi et al., 2014). All the presented pieces of evidence illustrate that the EnSCs could serve as a proper target for achieving an effective myogenic response in the ischemic limb tissue like the MSCs (Figure 3).

The stressful conditions that form early after ischemia has been able to cause cellular death cascades in all limb cell populations as well as neurons. Recreating the degenerated PNS within patients with a large limb injury could be an impactful method for recovering their lost limb functions. This cell therapy research has been focusing on finding a suitable source of stem cells to regenerate the PNS after limb ischemia. Considering the ethical grounds, purity, viability, and tumorigenicity, EnSCs can potentially be a promising source of easily accessible, substantial, and multipotent adult stem cells. In this endeavor, the EnSCs could be used as an alternative source (Figure 3). It is well understood that human EnSCs have provided new and effective approaches for neural cell programming. Our experiments reported that neuron-like cells were differentiated from EnSCs by different *in vitro* methods (Navaei-Nigjeh et al., 2014; Mirzaei et al., 2016; Hasanzadeh et al., 2021).

During the course of their research, Meng et al. (2007) have shown that the EnSCs, during their neurogenic differentiation, could express cytoskeletal proteins that include neurofilament-light (NF-L), class III b-tubulin (b3-tub), microtubule associated protein 2 (MAP 2), and oligodendrocyte transcription factor 1 (olig1) as mature markers in the differentiated neuronal-like cell (Meng et al., 2007). Moreover, in 2012, we were able to successfully make neuronal-like cell phenotypes from the EnSCs. Our flow cytometric analysis demonstrated the positive expression of cell surface markers including CD90, CD105, OCT4, CD44, and negative for CD31, CD34, and CD133. Moreover, our q-PCR and immunocytochemistry evaluation shown expression of the nestin, c-aminobutyric acid (GABA), MAP2, class III β-tubulin (b3-tub), and neurofilament light polypeptide (NF-L) in the EnSCs (Mobarakeh et al., 2012). Following our subsequent projects, Ebrahimi-Barough et al. (2013a) could generate oligodendrocyte progenitor cells (OPCs) from human EnSCs and Asmani et al. (2013) differentiated the OPCs in a 3D culture system (fibrin hydrogel) from EnSCs (Ebrahimi-Barough et al., 2013a; Ebrahimi-Barough et al., 2015). Due to these results, we hypothesized that the activation of the miR-338 cascade plays a central role in the generation of oligodendrocyte cells from the EnSCs (Ebrahimi-Barough et al., 2013b).

To further our studies, we carefully investigated and compared the differentiation potential of the BMMSC and EnSCs to motor neuron-like cells on a nanofibrous scaffold (poly ε-caprolactone) using signaling molecules. Immunostaining and real-time PCR demonstrated expression of beta-tubulin III, islet-1, motor neuron and pancreas homeobox 1 (MNX1), neurofilament-H (NF-H), paired box 6 (Pax6), and choactase-positive motor neurons. We finally concluded that both cells had potential in the differentiation of motor neuron-like cells, but EnSc was superior to BM-MSC (Shirian et al., 2016). During the course of the study, our team was able to differentiate Schwann cells from EnSCs for the first time, using fibrin gel as a 3D culture environment by induction media. Immunocytochemistry confirmed the indicative markers (S100 and P75) for Schwann cells (Bayat et al., 2016). Also, our more recent *in vivo* examination of animal models with PNS disorders (sciatic nerve injury) demonstrated nerve regeneration in rats after the sciatic transaction followed by human EnSCs treatment in a defined nanofiber conduit (Mohamadi et al., 2018). As a result, the motor and sensory integrity were significantly improved in EnSCs-treated animals. All the pieces of evidence given before are able to display the feasibility and efficacy of the EnSC therapy to regenerate the injured limb’s PNS networks (Figure 2).

## Challenges and Solutions

**FIGURE 4:**
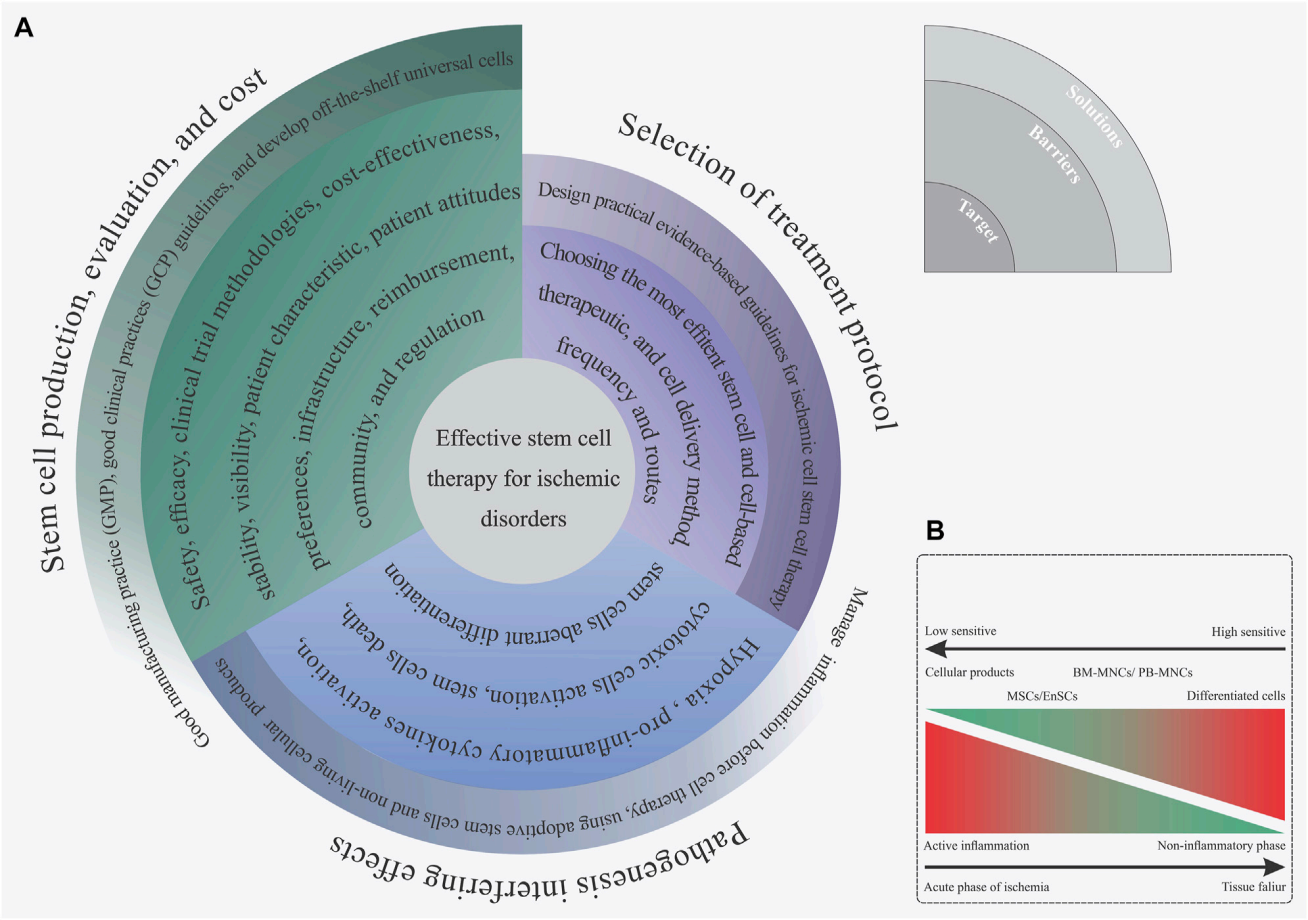
**(A)** Spiral diagram of challenges and solutions toward an effective stem cell therapy for ischemic disorder. **(B)** Complex biological outcome resulting from the ischemia inflammatory microenvironment on the implanted stem cells based on the stem cell type.

In an effort to develop a more efficient method of stem cell transfusion, scientists have spent more than 13 years doing clinical research on the subject (Lobo et al., 1991). After numerous observations, trial-and-error, and analysis, we have finally reached a breakthrough for stem cell-based therapy approaches, mainly for ischemic disorders. Proper understanding of the most common barriers and challenges is key to achieving an effective stem cell therapy method that could assist in the regeneration of ischemic regions. Based on our experience, these aforementioned barriers can be summarized into three separate steps: 1) stem cell manufacturing, 2) choosing the fittest stem cells based on disease characteristics, and 3) mechanisms of ischemia pathogenesis. In this section, we focus on these main challenges and then explain some practical solutions for overcoming those challenges. Figure 4 briefly displays the different limitations of ischemic disorder stem cell therapy and presents some efficient solutions for each issue (Figure 4).

### Stem Cells Manufacturing

Nowadays, developing technologies for the large-scale production of viable stem cells, and/or cellular products is a determining factor for the expansion of stem cell-based therapy in practice (Ausubel et al., 2011). Utilizing these types of technologies not only has a positive impact on reducing the price of cell therapy for each case, but can also be a solution to overcome ethical and logistical issues found with autologous stem cell therapies (Ausubel et al., 2011). Assuring good quality standards for biopharmaceutical production is done by following the good manufacturing practice (GMP) regulations (Jo et al., 2020). Accordingly, the GMP standards must be considered when manufacturing different cell products, including stem cells and differentiated cells. In this regard, GMP’s quality management system is largely used to monitor all the phases of the biopharmaceutical manufacturing process. Based on the GMP instructions, the purpose of the quality control (QC) section of a medical production factory is to guarantee product quality. It is based on a clear association between accurate assessment and critical quality features of the product of interest, including having an identity, being safe, pure, and potential (Viganò et al., 2018). Particular guidelines like the U.S. Food and Drug Administration (FDA) and European Medicines Agency (EMA) identify and explain the as-mentioned matters (Halme and Kessler, 2006; Emea, 2008). Some crucial steps in safety assessments are the evaluation of any microbial contamination (viral, bacterial, and fungal), teratogenic potential, and any biological abnormality in the final cell product (Viganò et al., 2018). These types of accurate quality controls and potentiometry of cell products make GMP guidelines a very powerful tool for the production of safe and efficient stem cells for a cost-effective stem cell therapy approach.

### Selection of an Efficient Stem Cell Type

Identifying and utilizing the most proper types of stem cell production, dosage, and implantation methods is another main issue for managing effective stem cell therapy in ischemic tissues. Achieving this mentioned target is directly related to having a suitable awareness of the ischemia intensity, phase of inflammatory reactions, reperfusion status, and several additional characteristics that appear in ischemic organs (Khodayari et al., 2019). After reviewing the collected information that can be found in Supplementary Table S1, it is hypothesized that this recorded controversial outcome, resulting from the stem cell administration, may be linked with a shortage of accurate awareness of the disease status and its possible feedback on the implanted cells. Nowadays, guideline-based medicine has become the mainstay of ischemia treatment (Zabel et al., 2020). Presenting these types of practical, evidence-based guidelines for stem cell therapy of ischemic disorders can be a solution to overcoming this challenge. The availability of clinical data, in particular from randomized controlled clinical trials (RCTs) for a specific query generally leads to the guidelines with overwhelming evidence (de Clercq, 2003). Concisely, the RCTs of ischemia stem cell therapy should meet inclusive and exclusive requirements. These criteria provide a wide and enriched study population, leading to expected goals along with different results from statistical and clinical points of view (Burns et al., 2011). Subgroup analyses on the predetermined ones and other post hoc analyses can assist with the identification of the features linked with more advantages, without benefit or damage from stem cell-based therapy, in the study population (Tanniou et al., 2016). So far, the FDA has released some guidelines for cellular and gene therapy approaches (Corsaro et al., 2021); however, none of them refer to ischemia disease treatments. Presenting an evidence-based guideline in order to administer the most efficient stem cell therapy treatment based on the patient’s characteristics can be an evolutionary achievement in the field of cell therapy for ischemic diseases.

### Ischemia Pathological Mechanism Adverse Effects

The process of ischemia pathogenesis explains a complex and dynamic biological mechanism that eventually terminates in organ familiarity and dysfunction, as we previously explained in section one, “limb ischemia pathophysiology: pathways and mechanisms.” In this regard, the formation of these types of complex biological reactions following ischemia disrupts the implanted stem cells (Khodayari et al., 2019). That means that the development of these mechanisms will not only promote cell death and tissue degeneration in the ischemic organ but can also have a negative biological effect on the implanted cells. In 2019, we hypothesized that the creation of an intense inflammatory response during the acute phase of myocardial ischemia can be a confounding factor in the reduction of efficacy for cell therapy in patients with acute myocardial infarction. Hence, employing types of efficient paraclinical testings in order to obtain an accurate recognition of the disease features and failure characteristics can be an effective approach in future cell therapy measures. In addition to this approach, reducing inflammation before cell therapy as well as stem cell concomitant injection with anti-inflammatory drug therapy or cells with immunoregulatory potential, like MSCs, can be an efficient approach to solving this challenge (Khodayari et al., 2019).

Instead of direct implantation of stem cells, it has been suggested that using extracellular vesicles (exosomes) can be a new perspective on regenerative medicine and cell-free therapy in ischemia subjects with active inflammation (Anderson et al., 2016; Liang X. et al., 2016; Phinney and Pittenger, 2017). This type of stem cell product is entirely resistant to inflammation pathogenesis (Xia et al., 2019). Direct cell-cell micro-communication is commonly mediated by exosomes through the use of critical functional molecules including nucleotides, proteins, as well as bioactive lipids. Different SCs, for instance, MSCs, secrete the as-mentioned small membrane vesicles (30–100 nm) (Anderson et al., 2016; Liang X. et al., 2016; Phinney and Pittenger, 2017). It is believed that healing after myocardial infarction might be regulated by MSCs-secreted exosomes. A research study was conducted on rat models with acute myocardial infarction (AMI) and proved that MSC-derived exosomes improve the function of the myocardium just after heart injury. This is related to inflammatory microenvironment reprogramming in AMI. Our *ex vivo* investigation in 2019 has suggested that the EnSCs-derived exosome could regulate angiogenesis and vessel formation in the endothelial progenitor cells (Taghdiri Nooshabadi et al., 2019). As a result, we found that this EnSCs-derived exosome angiogenic outcome operates by a dosage-dependent action (Taghdiri Nooshabadi et al., 2019), as evidenced by the dual results observed in drugs and agents as well as atorvastatin (Goodarzi et al., 2020). In summary, once EnSC-derived exosomes hold biological effects similar to their source cells, they could be deemed as new, cell-free, therapeutic candidates. Generally, as angiogenesis increases, the exosomes derived from EnSC indicate the possibility for use in regenerative medicine, particularly for ischemic disorders (Taghdiri Nooshabadi et al., 2019). So far, in addition to targeting treatment of acute ischemic stroke (NCT03384433), different clinical studies have been launched to evaluate the safety and efficacy of stem cell-derived exosomes on different disorders like acute respiratory distress syndrome (NCT04602104, NCT04798716), depression, anxiety, dementias (NCT04202770), periodontitis (NCT04270006), and metastatic pancreas cancer (NCT03608631).

## Conclusion and Future Perspective

Destruction of all cellular populations soon after the initiation of ischemia is an inevitable outcome in the ischemic limb regions. Recreation of vascular networks in a stressed tissue or organ is introduced as the first line of stem cell therapy for these patients. However, it appears that through neovascularization, improving blood circulation is not therapeutic enough to recover the ischemic limb function. Theoretically, utilization of a safe stem cell variety with the potential to generate other limb tissue’s cellular niches in addition to the endothelial cells, such as the myocytes and PNS cells, dramatically enhances the stem cell therapy effectiveness to promote the limb ischemia patient’s function. According to these criteria, although stem cell therapy has offered a closely acceptable output in various experimental and clinical trials, taking advantage of new and more proper stem cell sources will provide us with a suitable treatment for injured limb regeneration. Throughout the last decade, our lab’s efforts were to clarify the EnSCs’ biology and regeneration potential for improving several degenerative disorders. We remark that although the EnSC niche is derived from an inappropriate tissue, the high potential to generate an array of cellular populations alongside their low immunogenicity will make this lineage a new source of LI regeneration. Using this method, designing translational studies and then measuring the direct regenerative effects of EnSCs on the failed limb are one of our main approaches. However, different limitations and unsolved barriers prevent us from performing an effective regeneration therapy on ischemic diseases using life and autologous stem cells. Nonetheless, to overcome this challenge, researchers should move to develop modern approaches according to prior experiences.It seems to be an adequately safe, efferent, and cost-effective regeneration therapy method for different types of human disorders by: 1) designing a practical evidence-based guideline for stem cell administration, 2) utilizing next-generation regenerative methods, including off-the-shelf universal stem cell and cell-free exosome therapy, and 3) moderating the ischemic limb tissue inflammatory response before cell injection by using common therapeutics seems to be an adequately safe, efferent, and cost-effective regeneration therapy method for different types of human disorders.

## Abbreviations

**ANGPT2,** angiopoietin-2; **BDNF,** brain-derived neurotrophic factor; **BM-MNCs,** bone marrow mononuclear cells; **CCL-2,** C-C motif chemokine ligand 2; **CCL5,** C-C motif chemokine ligand 5; **CD34^+^ MNCs,** CD34-positive mononuclear cells; **C-Kit,** tyrosine protein kinase Kit; **CXCL1,** X-C motif chemokine ligand 1; **CXCL5,** X-C motif chemokine ligand 5; **CXCL8,** C-X-C motif chemokine ligand 8; **EGF,** epidermal growth factor; **ESCS,** endometrial stem cells; **ERC,** endometrial regenerative cells; **ESPCs,** endothelial stem/progenitor cells; **GATA2,** GATA-binding factor 2; **HLA-DR,** human leukocyte antigen—DR; **ANGPT2,** angiopoietin-2; **ICAM-1,** intercellular adhesion molecule 1; **IL-10,** interleukin-10; **KDR,** kinase insert domain receptor; **MSCs,** mesenchymal stem cells; **NMPB-ACPs,** non-mobilized peripheral blood angiogenic cell precursors; **NO,** nitric oxide; **NOS,** nitric oxide synthase; **IL-8,** interleukin-8; **PDGF,** platelet-derived growth factor; **PDGFB,** platelet-derived growth factor subunit B; **PGPC,** *Escherichia coli* K-12; **PROM1,** prominin 1; **SDF-1,** stromal cell-derived factor 1; **SGPC,** streptogrisin-C; **TGF-β,** transforming growth factor-β; **VCAM-1,** vascular cell adhesion protein 1; **VEGF,** vascular endothelial growth factor; **VEGFR,** vascular endothelial growth factor receptor. **GFAP,** glial fibrillary acidic protein; **SOX,** SRY (sex-determining region Y)-Box 1; **FOXO-3,** forkhead box O3; **TLX,** orphan nuclear receptor TLX; **IGF-1,** insulin-like growth factor-1; **GDNF,** glial cell-derived neurotrophic factor; **NGF,** nerve growth factor.
