# Supplementary material for: Stem Cell Therapy in Limb Ischemia: State-of-Art, Perspective, and Possible Impacts of Endometrial-Derived Stem Cells
Source: Front Cell Dev Biol. 2022 May 23;10:834754. doi: 10.3389/fcell.2022.834754 (PMC9168222; doi:10.3389/fcell.2022.834754)
Supplement: Supplementary file 1 [file Table1.docx]

| **Table S1:** Summary of some published and ongoing clinical trial on limb ischemia stem cell therapy according to the cell type. | | | | | |
| --- | --- | --- | --- | --- | --- |
| **Stem cell type** | **Study phase/ design** | **Participants/ Criteria** | **Stem cell source/ phenotype/ injection protocol** | **Main outcome/ conclusion** | ***Ref*** |
| **BM-MNCs** | **Phase:** I/II  **Study design:** randomized, double- blind, placebo-controlled trial.  **Aim of study:** investigate the “safety and efficacy of repeated BM- MNCs injections in comparison with a single BM-MNCs injection in CLI patients”. | **N=** 38  **Criteria:** Patients with atherosclerosis-related CLI. | **Source:** bone marrow  **Phenotype:** CD34^+^  **Treatment protocol:** 30 IM injection with approximately 1 mL of BM-MNC suspension into each injection site. | · Indexes of the secondary endpoints including pain, ulcers, TcPO2, and ankle-brachial index value were not statistically improved.      · Injection of the BM-MNCs in CLI patients decreased the risk of major amputation in patients showing with nonrevascularizable. | ([Pignon et al., 2017](#_ENREF_126)) |
|  | **Phase:** I/II  **Study design:** pilot randomized controlled trial  **Aim of study:** investigate the “safety and efficacy of repeated BM- MNCs injections in comparison with a single BM-MNCs injection in CLI patients”. | **N=** 22  **Criteria:** presented with atherosclerosis-related CLI. | **Source:** bone marrow  **Phenotype:** CD34^+^, CD133^+^, VEGFR^+^, VWF^+^, and TIE2^+^  **Treatment protocol:** 40–60 IM injection with 2-4×10^6^ MSCs per ml in each injection. | · Following cell therapy, researchers could record a significant increase in Ankle-Brachial Index, Visual Analog Scale, pain-free walking distance, Wagner stage, and also reduction in ulcer size.    · An interesting long- term advantage from the transplanted BM-MNCs was observed even in the patients who were treated with a single injection.    · Repeated cell transplantation had a higher impact on the improvement of pain-free walking distance. | ([Molavi et al., 2016](#_ENREF_113)) |
|  | **Phase:** III  **Study design:** randomized, placebo controlled, double blind clinical trial.  **Aim of study:** “evaluate whether the improved QoL persisted beyond 6 months' follow up, whether this differed in both trial arms”. | **N=** 109  **Criteria:** presented with no option severe limb ischemia. | **Source:** bone marrow  **Phenotype:** CD34^+^  **Treatment protocol:** administration of 10 ml BM-MNC into the common femoral artery of the affected leg. | · “The increased QoL in patients with no option severe limb ischemia persisted until 3 years after inclusion, but did not differ between the BMMNC and placebo arms or between patients with and without a major amputation”. | ([Weem et al., 2016](#_ENREF_165)) |
|  | **Phase:** II  **Study design:** randomized controlled trial  **Aim of study:** “therapeutic effect of G-CSF administration following implantation of autologous BM-MNC for patients with lower LI”. | **N=** 15  **Criteria:** presented with severe chronic limb ischemia | **Source:** bone marrow  **Phenotype:** TIE2^+^, VEGFR2^+^, VWF^+^, CD133^+^, and CD34^+^  **Treatment protocol:** “approximately  10 × 10 ^8^ cells were injected in normal saline supplemented with 2% patient serum”. | · In comparison with baseline, several clinical indexes including ankle-brachial index, visual analog scale, and pain-free walking distance were improved.  · There was no significant variation in cell therapy outcome in the MNC – G-CSF and MNC G-CSF groups.  . No severe adverse reactions were observed flowing the study. | ([Zafarghandi et al., 2010](#_ENREF_178)) |
| **MSCs** | **Phase:** I-b  **Study design:** nonrandomized, uncontrolled, open-label, dose- escalation phase study.  **Aim of study:** “effects of karyotype abnormalities on efficacy of MSCs for “no-option” CLI treatment”. | **N=** 12  **Criteria:** patients with no-option CLI | **Source:** bone marrow  **Phenotype:** CD73^+^, CD90^+^, CD105^+^, cocktail^-^, CD45^-^, CD34^-^, CD11b^-^, CD19^-^, and HLA-DR^-^  **Treatment protocol:** IM injection of 20×10^6^ - 40×10^6^ MSCs | · There were no notable improvements resulting from the MSCs injection into CLI patients.  · "The results of this trial concluded that an autologous cell therapy approach with MSCs for CLI is limited by the high rate of karyotype abnormalities". | ([Mohamed et al., 2020](#_ENREF_112)) |
|  | **Phase:** II  **Study design:** single-center, double-blinded, randomized, placebo-controlled trial.  **Aim of study: “**long-term outcomes of BM-MSC compared with BM- MNC for treatment CLI” | **N=** 41  **Criteria:** patients with bilateral CLI and foot ulcer. | **Source:** bone marrow  **Phenotype:** CD29^+^, CD71^+^, CD90^+^, CD105^+^, CD45^-^, and CD34^-^  **Treatment protocol:** IM injection of 9.3 ± 1.1 × 10^8^ MSCs | · Flowing 3-6 months after the BM-MSCs transplantation, a significant improvement in ulcer healing and recurrence rate was demonstrated in compared with BM-MNCs received groups.  . Injection of BM-MSC had a greater time of limb salvage and blood flow improvement within 9 months in compared with the BM-MNCs treated group. | ([Lu et al., 2019](#_ENREF_103)) |
|  | **Phase:** II  **Study design:** double-blind, placebo-controlled randomized clinical trial.  **Aim of study:** effect of “allogeneic BM-MSCs in patients with CLI who are not eligible for revascularization”. | **N=** 66  **Criteria:** patients with no-option critical ischemic limbs. | **Source:** bone marrow  **Phenotype:** CD29^+^, CD71^+^, CD90^+^, CD105^+^, CD45^-^, and CD34^-^  **Treatment protocol:** 30 IM injection with 5×10^6^ MSCs per injection. | · IM allogeneic BM-MSC therapy for CLI was safe and probably effective. | ([Wijnand et al., 2018](#_ENREF_166)) |
|  | **Phase:** II  **Study design:** multicenter, double- blind, placebo-controlled randomized clinical trial.  **Aim of study:** assess the “efficacy and safety of IM injection of BMMSC in CLI due to Buerger’s disease”. | **N=** 90  **Criteria:** patients with no-option critical ischemic limbs Buerger’s disease. | **Source: bone** marrow  **Phenotype:** CD73^+^, CD90^+^, CD105^+^, CD166^+^, CD45^-^, CD34^-^, CD133^-^, CD19^-^ and HLA-DR^-^  **Treatment protocol:** 40–60 IM injection with 2-4×10^6^ MSCs per ml in each injection. | · Significant improvement in the primary endpoints (reduction in rest pain and healing of ulcers) and secondary endpoints (ankle brachial pressure index and total walking distance) was observed in the group receiving 2×10^6^ cells/kg dosage.  · “IM administration of BM-MSC at a dose of 2×10^6^ cells/kg showed clinical benefit and may be the best regimen in patients with CLI due to Buerger’s disease”. | ([Gupta et al., 2017](#_ENREF_62)) |
| **PB- CD34^+^ MNCs** | **Phase:** III  **Study design:** retrospective, single- centered consecutive cohort  **Aim of study: “**identify the prognostic factors of responders and develop a predictive nomogram to guide patient selection flowing the cell therapy”. | **N=** 103  **Criteria:** presented with no option severe limb ischemia. | **Source:** G-CSF mobilized peripheral blood stem cells.  **Phenotype:** CD34^+^  **Treatment protocol:** IM injection of 10^5^-10^6^ cell/kg into the ischemic limbs *via* equidistant IM injections (0.5 mL/site) under general anesthesia. | . As the main findings patients' age, blood fibrinogen, arterial occlusion level, TcPO2, and the total transplanted CD34^+^ cell have the main role in the determination of the cell therapy outcome. | ([Pan et al., 2019](#_ENREF_120)) |
|  | **Phase:** II  **Study design:** prospective phase II clinical trial  **Aim of study:** “perspectival evaluation effects of G-CSF mobilized PB- CD34^+^ MNCs in presents with CLI undergoing hemodialysis”. | **N=** 6  **Criteria:** presents with CLI undergoing hemodialysis. | **Source:** G-CSF mobilized peripheral blood stem cells.  **Phenotype:** CD34^+^  **Treatment protocol: “**all CD34^+^ cells dissolved in 10 ml physiological saline were administered intramuscularly into 40 sites (0.25 ml per site) of the leg with more severe ischemia”. | · Some main clinical indexes including the Fontaine scale and Rutherford category dramatically improved 24-52 weeks after cell transplantation.  · One year flowing the cell therapy imputation-free survival rate was 100%.    · G-CSF-mobilized CD34^+^ MNCs therapy was safe, feasible, and effective for the CLI patients. | ([Ohtake et al., 2018](#_ENREF_119)) |
|  | **Phase:** II  **Study design:** prospective phase II clinical trial.  **Aim of study:** “evaluate the long- term safety and efficacy of IM transplantation of GCSF-mobilized CD34^+^ cells in no-option patients with CLI for up to four years post cellular therapy”. | **N=** 17  **Criteria:** presents with CLI with atherosclerotic peripheral arterial disease and Buerger's disease. | **Source:** G-CSF mobilized peripheral blood stem cells.  **Phenotype:** CD34^+^  **Treatment protocol:** “CD34^+^ cells dissolved in 10 ml saline were intramuscularly injected into 40 sites at totally 10^5^- 5×10^5^ cells/kg dosage”. | · The G-CSF-mobilized CD34^+^ MNCs therapy had a favorable outcome for improvement of the brachial pressure index and transcutaneous partial oxygen pressure.    · Also, long-term improvement in the patient's physiological evidences was clearly shown the benefit of GCSF-mobilized CD34^+^ MNCs transplantation for patients with CLI, especially in the Buerger's disease cases. | ([Kinoshita et al., 2012](#_ENREF_90)) |
| **ESPCs/ NMPB-ACPs** | **Phase:** II  **Study design:** single center randomized unblended clinical trial  **Aim of study: “**compare the safety and therapeutic effects of enriched circulating EPCs with BM-MNC administration”. | **N=** 40  **Criteria:** presents with CLI | **Source:** non-mobilized peripheral blood cells  **Phenotype:** CD14^+^/CD34^low^  **Treatment protocol:** IM injection of 30×10^6^ - 840×10^6^ cells/kg dosage. | · A definite association between the administrated CD14^+^CD34^low^ cell dosage and the increase in muscle perfusion was observed.    · In both cell therapy groups, the rate of deaths and major amputations reduced in comprehension with baseline.    · "This study supports previous trials showing the efficacy of BM-MNC auto-transplantation in CLI patients and demonstrates comparable therapeutic efficacy between BM-MNC and EPEPCs". | ([Liotta et al., 2018](#_ENREF_100)) |
|  | **Phase:** I  **Study design:** pilot non- randomized, open-label study.  **Aim of study:** Assess the “efficacy and safety of the implantation of NMPB-ACPs in patients with CLI who were poor candidates for standard revascularization treatment options. | **N=** 6  **Criteria:** presents with CLI who were poor candidates for standard revascularization treatment options. | **Source:** non-mobilized peripheral blood cells  **Phenotype:** CD31^low^/CD34^low^  **Treatment protocol: “**54.5 ×10^6^-  10.2 ×10^6^ cell were administered by thirty IM injections into the gastrocnemius muscle of the ischemic limb”. | · The adequate circulation at the distal limb for complete healing was significantly improved in most of the patients.  · Also, complete regeneration of the ischemic ulcers and margins of toe amputation had been reported in any subjects.    · Administration of the NMPB-ACPs was safe and effective in the CLI patients. | ([Mutirangura et al., 2009](#_ENREF_117)) |
|  | **Phase:** I/II  **Study design:** single group assignment study.  **Aim of study: “**Assess the autologous immunomagnetic selected CD133^+^ EPCs in the treatment of no-option CLI”. | **N=** 8  **Criteria:** presents with CLI without revascularization options. | **Source:** peripheral blood  **Phenotype:** CD133^+^  **Treatment protocol:** 45-48 ml of autologous CD133^+^ saline solution suspension is administered intramuscularly in four separate limb margins. | · No results posted (ongoing study) |  |
|  | **Phase:** I/II  **Study design:** single group assignment.  **Aim of study:** study of the use of EPCs therapy in insulinized diabetic patient’s type 2 with critical ischemia in lower limbs. | **N=** 10  **Criteria:** insulinized diabetic patients type 2 with critical ischemia in lower limbs. | **Source:** bone marrow  **Phenotype:** CD133^+^  **Treatment protocol:** intra-arterial infusion of autologous CD133^+^ stem cells. | · No results posted (ongoing study) |  |
| **CTX (neural stem cell line)** | **Phase:** I  **Study design:** opened, randomized and controlled trial.  **Aim of study: “**safety study of IM CTX0E03 in patients with lower limb ischemia”. | **N=** 10  **Criteria:** patients with ischemia in lower limbs. | **Source:** CTX0E03 cell line  **Phenotype:** unknown  **Treatment protocol:** patients receive 10 IM injections of 20 ×10^6^- 80 ×10^6^ cell into the gastrocnemius muscle of their ischemic leg on a single occasion. | · No results posted (ongoing study) |  |
| **EnSCs** | **Phase:** I/II  **Study design:** opened, randomized and controlled trial.  **Aim of study: “**assessing the safety and feasibility of using ESCs in patients with CLI that are not eligible for surgical or catheter- based interventions”. | **N=** 15  **Criteria:** patients with CLI that are not eligible for surgical or catheter- based interventions | **Source:** menstrual mesenchymal stem cells (otherwise known as Endometrial Regenerative Cells, or ERC)  **Phenotype:** unknown  **Treatment protocol:** Patients will be treated with either 25 million, 50 million, or 100 million ERC by IM injection. | · No results posted (ongoing study) |  |
| **Abbreviations**  **BM-MNCs**: Bone marrow-derived mononuclear cells; **CLI:** critical limb ischemia; **ESCs:** Endothelial progenitor cells; **ESPCs:** Endothelial stem/progenitor cell; **G-CSF:** granulocyte colony–stimulating factors; **HLA-DR:** Human Leukocyte Antigen – DR; **IM:** Intramuscular; **MSCs:** Mesenchymal stem cell; **NMPB-ACPs:** Non-mobilized peripheral blood angiogenic cell precursors; **NSCs:** Neural stem cells; **PB- CD34^+^ MNCs:** Peripheral blood-derived CD34 positive mononuclear cells; **QoL**: Quality of life; **TcPO2**: Transcutaneous oximetry; **TIE2**: TEK tyrosine kinase 2; **VEGFR:** Vascular endothelial growth factor receptor; **VWf:** von Willebrand factor. | | | | | |
